# Seropositive Prevalence of Antibodies Against SARS-CoV-2 in Wuhan, China

**DOI:** 10.1001/jamanetworkopen.2020.25717

**Published:** 2020-10-23

**Authors:** Anding Liu, Ying Li, Zhengce Wan, Wenjie Wang, Xiaomei Lei, Yongman Lv

**Affiliations:** 1 Experimental Medicine Center, Tongji Hospital, Tongji Medical College, Huazhong University of Science and Technology, Wuhan, China; 2 Health Management Center, Tongji Hospital, Tongji Medical College, Huazhong University of Science and Technology, Wuhan, China

## Abstract

This cross-sectional study examines the seropositive prevalence of antibodies against severe acute respiratory syndrome coronavirus 2 (SARS-CoV-2) in Wuhan, China, by sex and age group.

## Introduction

A large number of individuals with coronavirus disease 2019 (COVID-19) infections might present with no or only mild symptoms, and the reported numbers of patients with COVID-19 do not reflect the true scale of the outbreak.^1^^,^^2^ Therefore, population-based serological studies are urgently needed to understand the epidemiological characteristics of the outbreak and the population’s immunity to COVID-19.

## Methods

This cross-sectional study was conducted in Tongji Hospital of Huazhong University of Science and Technology between March 27 and May 26, 2020. This study was approved by the ethics committee of Tongji Hospital of Huazhong University of Science and Technology. Informed consent was waived because deidentified data were used. This study follows the Strengthening the Reporting of Observational Studies in Epidemiology (STROBE) reporting guideline.

Adult participants aged 18 years or older were enrolled in the current study. None of the participants had a history of COVID-19. Demographic data, including age, sex, and residential region, were collected. The participants were screened for severe acute respiratory syndrome coronavirus 2 (SARS-CoV-2) infection by serological tests for IgM and IgG antibodies to SARS-CoV-2^3^ and by real-time reverse transcriptase–polymerase chain reaction tests for SARS-CoV-2 RNA.^4^ Additional details about the methods, including information on the statistical analysis, are provided in the eAppendix in the Supplement.

## Results

A total of 35 040 individuals (17 269 men [49.3%] and 17 771 women [50.7%]) were enrolled in this study. The median (interquartile range) age was 36 (30-45) years. The positivity rate for IgM antibodies only was 0.0%, that for both IgM and IgG antibodies was 0.7%, and that for IgG antibodies only was 3.2%. Most individuals (1100 of 1360 individuals [80.9%]) tested positive for IgG antibodies only. The overall seropositivity rate was 3.9% (95% CI, 3.7%-4.1%). We observed that very few individuals (15 of 35 040 individuals [0.04%]) had detectable SARS-CoV-2 viral nucleic acid sequence and tested negative during their quarantine period, and none of their close contacts had positive nucleic acid test results. The seropositive prevalence in the urban districts was higher than that in the suburban and rural areas (4.4% [95% CI, 4.0%-4.8%] vs 2.9% [95% CI 2.3%-3.6%]; *P* < .001), demonstrating an urban to suburban gradient. Moreover, women had higher seropositive prevalence than did men (4.4% [95% CI, 4.1%-4.6%] vs 3.3% [95% CI, 3.1%-3.6%]; *P* < .001). We did observe that seropositive prevalence was associated with increasing age, with the highest rates among individuals aged 60 years and older (9.2% [95% CI, 7.1%-11.3%]; *P* < .001) (Table and Figure).

**Table.** SARS-CoV-2 Seropositive Prevalence in Different Age Groups

Age group, y	Patients, No.	Male, %	SARS-CoV-2 seropositive prevalence, patients, No. (%) [95% CI]
18-29	8163	44.5	232 (2.8) [2.5-3.2]
30-39	13 471	44.3	524 (3.9) [3.6-4.2]
40-49	7713	52.3	313 (4.1) [3.6-4.5]
50-59	4932	64.7	221(4.5) [3.9-5.1]
≥60	761	49.3	70 (9.2) [7.1-11.3]

Abbreviation: SARS-CoV-2, severe acute respiratory syndrome coronavirus 2.

**Figure.**
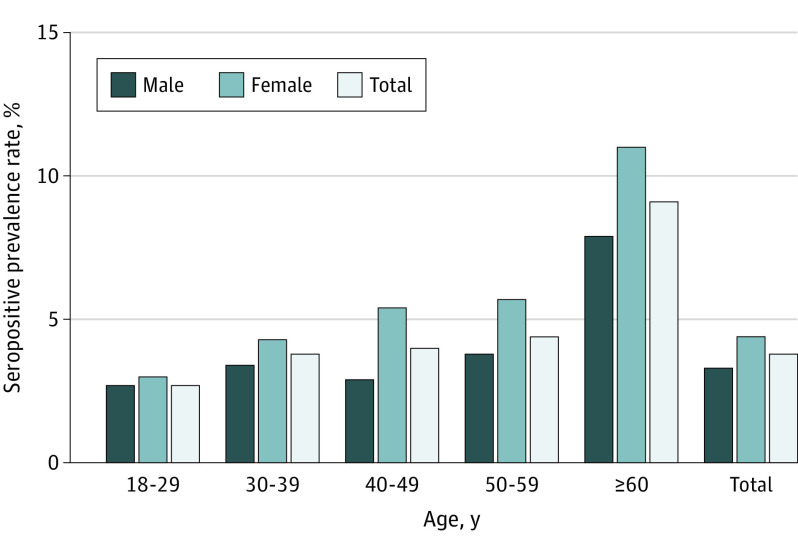
Severe Acute Respiratory Syndrome Coronavirus 2 Seropositive Prevalence by Sex and Age Group

## Discussion

This study found that the seropositive prevalence was 3.9% in a cohort of 35 040 individuals in Wuhan, China. Most individuals tested positive for SARS-CoV-2 IgG antibodies only, indicating a prior infection. We further showed that the seropositive prevalence in the urban districts was higher than that in the suburban and rural areas, which is consistent with the geographical distribution of confirmed cases, with the highest rates in the urban districts.^5^ Moreover, women had a higher seropositive prevalence than did men, which is consistent with a previous report^5^ showing that female individuals had higher rates of confirmed cases compared with male individuals. The seropositive prevalence was also significantly higher among elderly individuals than in other age groups. It is possible that elderly people had a higher proportion of comorbid conditions, which might facilitate SARS-CoV-2 infection and increase the severity of COVID-19.^5^

Our study has several limitations. Although the overall sample size was large, there were few participants older than 60 years and none of the participants were younger than 18 years, which limited our ability to estimate seropositive prevalence among elderly people and children. Because most of the participants came from urban districts with higher infection rates, seropositive prevalence may not be accurate. Because the specific antibodies against SARS-CoV-2 might wane over time in some convalescent COVID-19 individuals,^6^ asymptomatic cases who had low levels of antibodies might be more likely to become negative in population-based studies.

## Notes

Supplement. **eAppendix.** Supplemental Methods **eReferences**
