# Supplementary material for: Seropositive Prevalence of Antibodies Against SARS-CoV-2 in Wuhan, China
Source: JAMA Netw Open. 2020 Oct 23;3(10):e2025717. doi: 10.1001/jamanetworkopen.2020.25717 (PMC7584925; doi:10.1001/jamanetworkopen.2020.25717)
Supplement: Supplement. — eAppendix. Supplemental Methods eReferences [file jamanetwopen-e2025717-s001.pdf]

Supplemental Online Content

Liu A, Li Y, Wan Z, Wang W, Lei X, Lv Y. Seropositive prevalence of antibodies against SARS-CoV-2 in Wuhan, China. *JAMA Netw Open*. 2020;3(10):e2025717.
doi:10.1001/jamanetworkopen.2020.25717

eAppendix. Supplemental Methods
eReferences

This supplemental material has been provided by the authors to give readers additional information about their work.

© 2020 Liu A et al. *JAMA Network Open.*

eAppendix. Supplemental Methods

Subjects and sampling

This cross-sectional study was conducted in the Health Management Center of Tongji Hospital of Huazhong University of Science and Technology between March 27 and May 26, 2020. These participants were healthy community residents and employees who underwent SARS-CoV-2 nucleic acid test of nasal swabs, chest computed tomography (CT) scan and SARS-CoV-2 specific serological tests. None of the participants had a history of COVID-19. Finally, a total of 35,040 adult participants aged 18 years or older were enrolled in the current study. The time period of the sampling was after the first pandemic wave of COVID-19 in Wuhan, and sampling started about 8 weeks after the peak of the COVID-19 epidemic (January 23 to February 1).[1] Demographic data, including age, sex, and residential region, were collected. The participants were screened for SARS-CoV-2 infection by serological tests for IgM and IgG antibodies to SARS-CoV-2,[2] and by real-time reverse transcriptase–polymerase chain reaction (RT-PCR) tests for SARS-CoV-2 RNA.[3] This study was approved by the Ethics Committee of Tongji Hospital of Huazhong University of Science and Technology. Informed consent was waived because deidentified data were used. This study follows the Strengthening the Reporting of Observational Studies in Epidemiology (STROBE) reporting guideline.

Laboratory measurements

Nasal swab specimens were obtained to test for SARS-CoV-2 by real-time RT-PCR. Total RNAs from nasal swab specimens was extracted by a viral nucleic acid kit (Tianlong Science & Technology, Xian, China). The presence of SARS-CoV-2 was

© 2020 Liu A et al. *JAMA Network Open.*

detected by real-time RT-PCR amplification of SARS-CoV-2 open reading frame 1ab (ORF1ab) and nucleocapsid protein (NP) genes fragments (DAAN GENE, Guangzhou, China) as described elsewhere.[3] A cycle threshold (Ct)-value less than 40 was defined as a positive test.

Serum samples were isolated and stored at 4°C, and IgM and IgG antibodies to SARS-CoV-2 were measured within 24 hours. The IgM and IgG antibody against SARS-CoV-2 in serum samples were measured using a commercially available magnetic chemiluminescent immunoassay assay (MCLIA) kits (Bioscience, Tianjin, China), as described elsewhere.[2] Briefly, recombinant antigens contained the nucleoprotein and a peptide from the spike protein of SARS-CoV-2 were immobilized on magnetic particles. The anti-human IgG/IgM antibody-conjugated with alkaline phosphatase was used as the detection antibody. The IgM and IgG antibodies levels are presented as the chemiluminescence signal values divided by the cutoff (absorbance/cutoff, S/CO), and was considered a positive test when S/CO level was greater than 1. The sensitivity, specificity, positive predictive value, and negative predictive value of IgM antibody were 88.3%, 99.5%, 99.2% and 92.4%, respectively. The sensitivity, specificity, positive predictive value, and negative predictive value of IgG were 87.2 %, 99.3%, 98.8% and 91.7%, respectively.

Analysis

All statistical analyses were performed using SPSS 20.0. The 95% CI of the seroprevalence was calculated from binomial probabilities using Wilson's methods. Chisquare test was used for comparison of seroprevalence between groups, and logistic

© 2020 Liu A et al. *JAMA Network Open.*

regression analysis was performed to analyze the association between the seroprevalence and age, sex and region of residence.

eReferences

1. Pan A, Liu L, Wang C, et al. Association of Public Health Interventions With the Epidemiology of the COVID-19 Outbreak in Wuhan, China. *Jama.* 2020;323(19):1-9.
2. Long QX, Liu BZ, Deng HJ, et al. Antibody responses to SARS-CoV-2 in patients with COVID-19. *Nat Med.* 2020;26(6):845-848.
3. Chen X, Zhao B, Qu Y, et al. Detectable serum SARS-CoV-2 viral load (RNAaemia) is closely correlated with drastically elevated interleukin 6 (IL-6) level in critically ill COVID-19 patients. *Clin Infect Dis.* 2020.

© 2020 Liu A et al. *JAMA Network Open.*
